# Cardiac MR function analysis with DL-based super resolution reconstruction: application in the clinical setting

**DOI:** 10.1007/s10554-026-03642-8

**Published:** 2026-02-09

**Authors:** Franziska Adomat, Christof Schaub, Tobias Hoh, Xenia Fischer, Roman Guggenberger, Robert Manka, Matthias Eberhard, Lucas Weber

**Affiliations:** 1 Department of Radiology and Nuclear Medicine, Cantonal Hospital Winterthur, Winterthur, Switzerland; 2 Institute for Diagnostic, Interventional and Pediatric Radiology, University Hospital of Bern, Bern, Switzerland; 3 MR Clinical Science, Philips AG, Horgen, Switzerland; 4 Office for Sport and Health Promotion, Health Department of the Canton of Zug, Steinhausen, Switzerland; 5 Department of Cardiology, University Heart Center, University Hospital Zurich, Zurich, Switzerland; 6 Institute for Diagnostic and Interventional Radiology, University Hospital Zurich, Zurich, Switzerland

**Keywords:** Cardiac MRI, Cine imaging, Compressed SENSE, Deep learning, Super-Resolution, Reconstruction algorithm

## Abstract

To assess differences in volumetry, image quality and acquisition time between balanced steady-state free precession cine sequences acquired using (a) a standardized sensitivity encoding (SENSE) approach and (b) deep learning-based super-resolution reconstruction based on high-resolution images acquired with compressed sensitivity encoding (C-SENSE). We retrospectively evaluated 31 consecutive patients (mean age 61.2 ± 13.1 years, 26% female (8/31) and 74% male (23/31)) undergoing cardiac magnetic resonance imaging (MRI) examinations to assess for the presence of ischemic and non-ischemic cardiomyopathies. Cine images were acquired using a 1.5T Philips Ingenia MRI scanner, with classic parallel imaging (SENSE) and compressed sensing (C-SENSE) accelerated acquisition techniques (*R* = 2 and *R* = 4, respectively). C-SENSE datasets were reconstructed using a deep learning-based denoising and super-resolution algorithm to enhance image resolution and quality (CS-SR). To evaluate cardiac function, manual left ventricular (LV) segmentation and volumetric analysis were performed on both datasets by two readers, who were blinded to the clinical data. Image quality was rated independently by three readers using Likert scales. Correlation between SENSE and CS-SR datasets with respect to LV volumetry was high (*r* = 0.98-1.00), with no significant differences found for end-diastolic volume (mean difference 0.04 ml, limits of agreement (LoA) -11.19 to 11.26 ml; *p* = 0.970) or end-systolic volume (mean difference 1.60 ml, LoA − 7.48 to 10.68 ml; *p* = 0.064). Overall subjective image quality was comparable (*p* = 0.061), with CS-SR offering better image sharpness at the cost of increased artifacts (*p* < 0.001 respectively). Image acquisition time was significantly accelerated with C-SENSE acquisition (SENSE: 411.1 ± 47.7 s, C-SENSE: 165.6 ± 21.5 s; *p* < 0.001). CS-SR shows promise in streamlining routine cardiac imaging by significantly shortening acquisition times, without impairing LV volumetric analysis, while preserving overall image quality and resolution.

**Supplementary Information:**

The online version contains supplementary material available at 10.1007/s10554-026-03642-8.

## Introduction

Cardiac magnetic resonance imaging (MRI) offers high spatial and temporal resolution for the evaluation of cardiac function, morphology, and contrast dynamics. Cardiac cine imaging is an integral part of cardiac MRI protocols in numerous guidelines worldwide [1–5], enabling the assessment of cardiac function with high reproducibility and reliability. The current standard approach for the evaluation of biventricular function is retrospectively electrocardiogram (ECG) gated two-dimensional segmented cine imaging, with balanced steady-state free precession (bSSFP) representing the method of choice [1, 6, 7]. The advantages of bSSFP are the high signal-to-noise ratio and the excellent contrast between myocardium and blood pool. A challenge with this approach relates to the requirement for repeated breath-holds for whole-heart coverage, resulting in a long acquisition time, and the need to compensate for artifacts induced by cardiac and respiratory motion [8, 9].

Over the last few decades, various reconstruction techniques have been developed to reduce image acquisition time. Parallel imaging techniques, such as sensitivity encoding (SENSE), utilize arrays of multiple receiver coils in parallel to reduce the scan time [10]. More recently, undersampling techniques such as compressed sensitivity encoding (C-SENSE) have enabled significant scan time acceleration in clinical practice by using pseudorandomized undersampling of the k-space during the relevant cardiac phase [11–14], often at the cost of image resolution. Nevertheless, higher-fold scan time accelerations have not yet found widespread use in routine clinical application, with some sources reporting reduced image quality, blurring of cardiac contours, and excessively long breath-holds [11, 15, 16].

The application of deep learning (DL) image reconstructions to compressed sensing (CS) acquisition schemes has the potential to overcome these issues [17–19]. DL-based algorithms can optimize the CS algorithm to allow further scan acceleration and enhance image sharpness by mitigating ringing artifacts [20–22]. The application of an advanced DL-based super-resolution (SR) reconstruction algorithm (Precise-Image-Net) in addition to a denoising filter (Adaptive-CS-Net) to higher-fold accelerated C-SENSE cine images (CS-SR) has shown significant potential for reducing the overall scan time while maintaining good image quality in prostate, musculoskeletal, and breast imaging [18, 23–29].

The purpose of this study was to assess if DL-based SR reconstruction allows for the acquisition of high-resolution images with shortened acquisition times per image slice and reasonable breath-hold durations. We assessed the differences in volumetry, image quality and acquisition time between bSSFP cine sequences acquired using (a) a standardized sensitivity encoding (SENSE) approach and (b) DL-based SR reconstruction based on accelerated cine images acquired with compressed sensitivity encoding (C-SENSE).

## Materials and methods

### Study population

This study was performed in accordance with the principles of the Declaration of Helsinki. Approval for clinical studies involving the assessment of commercially available MRI sequences in routine imaging was granted by the Cantonal Ethical Committee Zürich, Switzerland (BASEC ID: 2019 − 00259) on 04.07.2019. Informed consent requirements with respect to study participation and publication were fully adhered to.

**Fig. 1:**
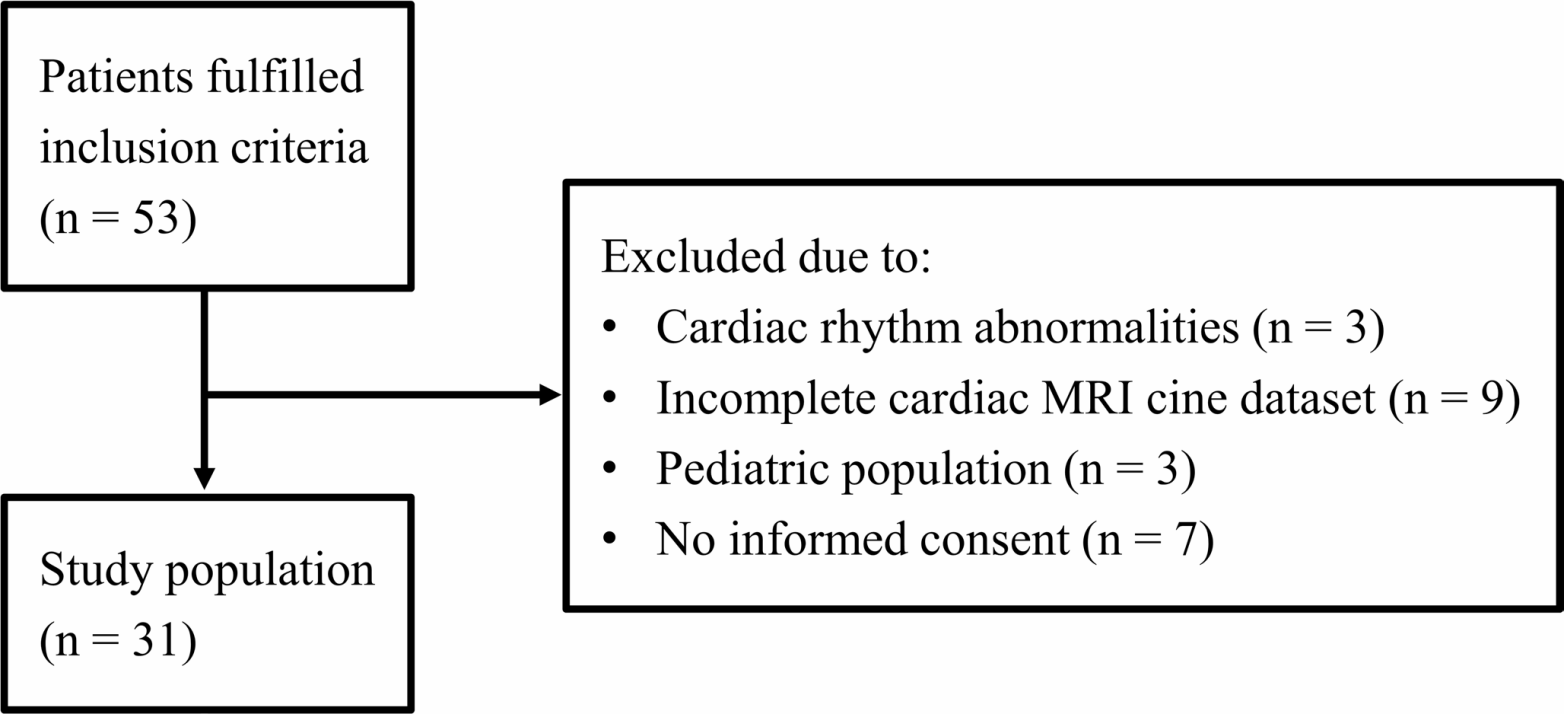
Study population

We retrospectively analyzed imaging data from patients that were consecutively scanned between August 2023 and November 2023. These patients underwent baseline or follow-up cardiac MRI examinations for both ischemic and non-ischemic cardiomyopathies at our institution (see Table 1). Patients were considered for inclusion only when the examination was performed on a scanner with the Super-Resolution software patch. Exclusion criteria were, (i) cardiac rhythm abnormalities, notably atrial fibrillation and ventricular extrasystoles, which could have a detrimental impact on image quality, (ii) age below 18 years, (iii) incomplete SAx cine imaging datasets, and (iv) declined or missing general consent. Thirty-one patients were ultimately included in the study as shown in Fig. 1.

**Table 1:**
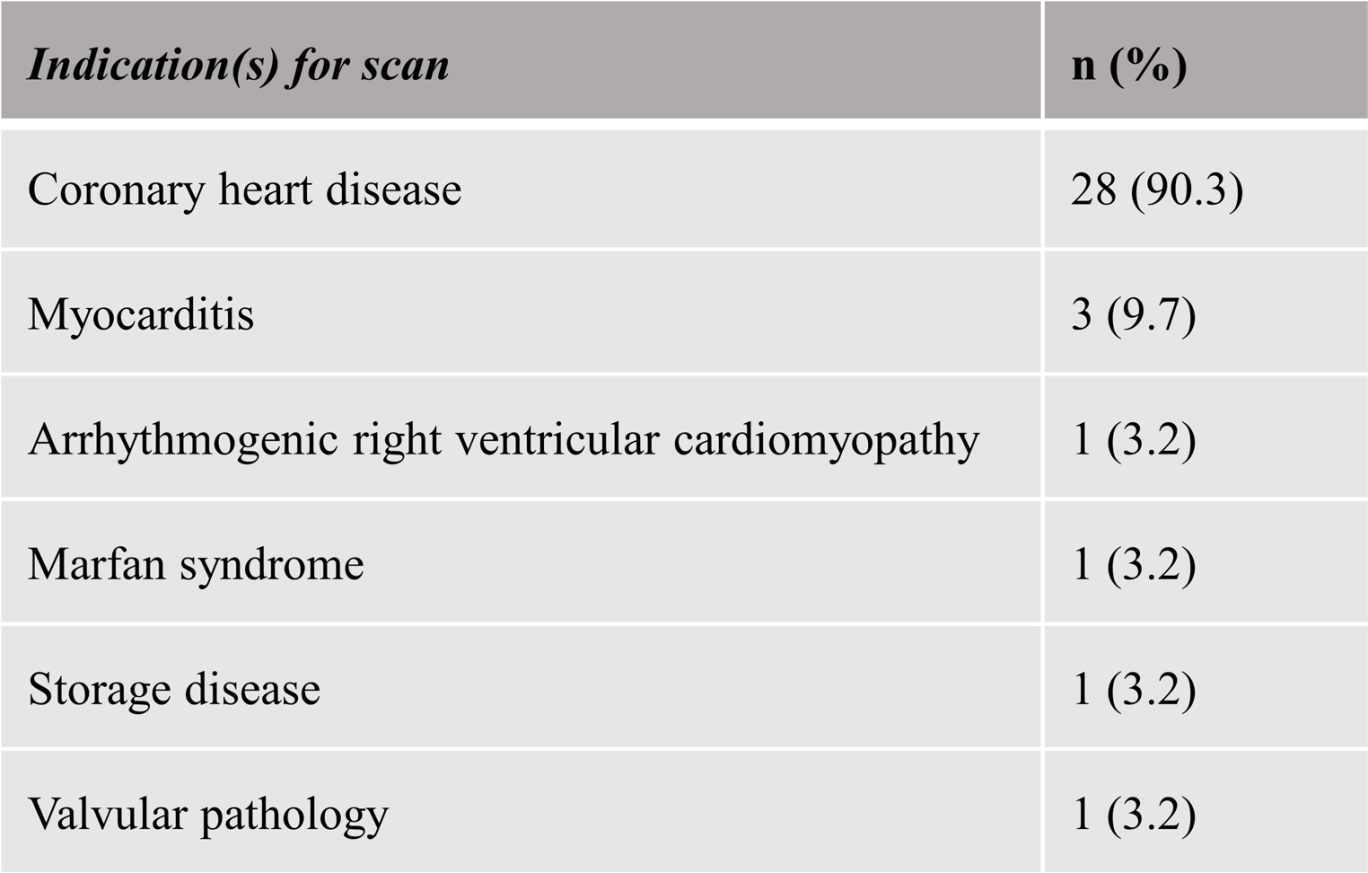
Indication(s) for scan. The indications for cardiac MRI included ischemic and non-ischemic cardiomyopathies. The number of clinical indications (n = 35) exceeds the patient number due to multiple indications in three cases

### Image acquisition

Cardiac cine images were acquired on a 1.5T Philips Ingenia MRI system (Philips Healthcare, Best, The Netherlands) using a 32-element receiver body coil array. Short-axis cine images with whole-heart coverage were acquired using a bSSFP sequence with SENSE and C-SENSE in one and three slices per breath-hold, respectively. Data were acquired at end-expiratory breath-hold to minimize respiratory motion artifacts. No data was shared between cardiac phases. The imaging protocol included standard long-axis views and short-axis (SAx) slices covering the entire left ventricle. SAx cine images were acquired before administration of the contrast agent.

SENSE imaging parameters were as follows: repetition time (TR): 2.6 ms, echo time (TE): 1.3 ms, spatial resolution: 1.7 × 1.7 × 8 mm, field of view (FOV): 300 × 300 × 96 mm, flip angle: 60°, bandwidth: 1100 Hz/pixel, acquired heart phases: 20, reconstructed heart phases: 30, undersampling factor *R* = 2. C-SENSE imaging parameters were as follows: TR: 2.7 ms, TE: 1.4 ms, spatial resolution: 1.7 × 1.7 × 8 mm, FOV: 300 × 300 × 96 mm, flip angle: 60°, bandwidth: 1100 Hz/pixel, acquired heart phases: 20, reconstructed heart phases: 30, undersampling factor *R* = 4.

Radiology technicians manually recorded the duration of image acquisition for each sequence, with the starting point defined as the manual start of image acquisition on the scanner. Duration of breath-holds was automatically determined by the scanner.

### Super-resolution reconstruction algorithm

**Fig. 2:**
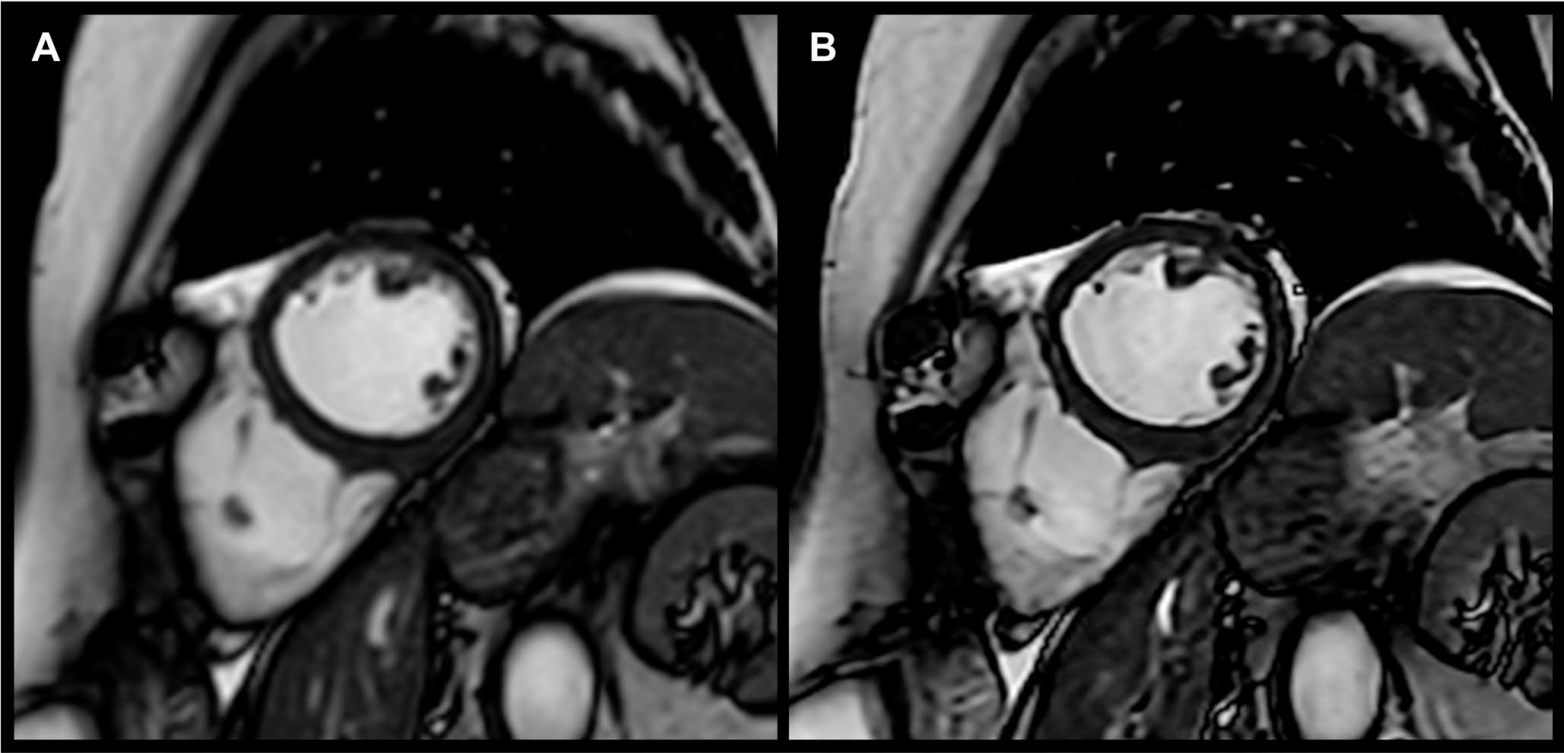
Direct comparison of end-diastolic left ventricular images in SAx, acquired using accelerated (**A**) SENSE at *R* = 2 and (**B**) C-SENSE at *R* = 4 with application of a denoising filter and SR reconstruction. The increased image sharpness at CS-SR is immediately apparent. Supplemental videos (1.1 and 1.2) are available for this case

SENSE and C-SENSE images were reconstructed using the vendor-provided online reconstruction. Additional reconstruction of the C-SENSE datasets was performed using a vendor-developed (Philips Healthcare, Best, The Netherlands) reconstruction algorithm that combines sparsity-constrained DL compressed sensing (Adaptive-CS-Net) and DL-based anti-ringing reconstruction (Precise-Image-Net) (see Fig. 2). These networks have both been previously established, with the combination of these two networks most notably reported by Bischoff et al. [18, 20, 21, 27–32].

### Left ventricular segmentation

To evaluate cardiac function, manual segmentation of the left ventricular (LV) endocardium was performed on SENSE and CS-SR cine images at end-systole and end-diastole, in accordance with standardized image interpretation [33]. The segmentation was conducted using specialized software (IntelliSpace Portal, Version 12.1, Philips Healthcare) by two board-certified radiologists (F.A. and C.S., with three and five years of experience in the evaluation of cardiac magnetic resonance (MR) images, respectively), who were blinded to the sequences viewed.

### LV volumetric analysis

Volumetric parameters such as the end-diastolic volume (EDV) and end-systolic volume (ESV) of the left ventricle were compared between the SENSE and CS-SR datasets.

### Assessment of image quality

Visual image quality assessment was carried out independently by three radiologists (F.A., C.S. and L.W., with three, five and six years of experience reading cardiac MR images, respectively), who were blinded to the sequences viewed and to all identifying patient information. The readers evaluated overall image quality (general image impression), image sharpness, and the presence of artifacts (including motion, ringing, partial volume, and susceptibility artifacts). A five-point Likert scale was used to assess each category, with the results being averaged across the three readers. The Likert scales for the overall image quality, and the image sharpness were marked as follows: excellent (5), good (4), moderate (3), fair (2), poor (1). The Likert scale for the presence and severity of artifacts was denoted as: none (5), mild (4), moderate (3), marked (2), non-diagnostic (1).

Further image analysis was conducted by calculating the apparent signal-to-noise ratio (signal intensity in the myocardium divided by the standard deviation of the blood-pool) and apparent contrast-to-noise ratio ([signal intensity of the myocardium minus signal intensity of the blood-pool] divided by the standard deviation of the blood-pool) of the two datasets, with measurements obtained at comparable regions of interest during LV diastasis. Additionally, the contrast signal between the myocardium and the blood-pool ([signal intensity of the blood-pool minus signal intensity of the myocardium] divided by [signal intensity of the blood-pool plus signal intensity of the myocardium]) was calculated for both datasets, with a value of 1 indicating the maximum possible contrast and a value of 0 indicating no contrast.

### Statistical analysis

Population characteristics are presented as the means and standard deviations for continuous variables as well as counts and percentages for categorical variables. The Student’s t test for paired samples was used to compare volumetric data points EDV and ESV for SENSE and CS-SR. Bland-Altman diagrams were used to graphically illustrate the agreement between the two measurement methods and to assess for potential bias. Bland-Altman plots were calculated for the entire patient cohort, as well as a subset of patients with reduced LVEF (< 54%). The limits of agreement (LoA) were defined as ± 1.96 SD from the mean difference.

Interobserver reliability was assessed in a randomly chosen subset of ten participants, measured by use of the Pearson correlation coefficient.

For visual evaluation in all categories (overall image quality, artifacts, image sharpness) to evaluate interrater agreement intraclass correlation coefficients (ICC) were calculated in each category using ANOVA. To compare Likert scale ratings, a mean score was calculated in each category, with Student’s t-test for paired samples being used to compare data points.

## Results

### Patient characteristics

We included 31 patients, of which 23 were male (74%) and 8 were female (26%), with a mean age of 61.2 ± 13.1 years (range: 21–84 years). There was no significant difference between the patients’ heart rates during the acquisition of SENSE vs. C-SENSE cine images (SENSE 67 ± 13 bpm, C-SENSE 69 ± 13 bpm, *p* = 0.59).

One patient did not complete the entire MRI examination due to claustrophobia and severe fatigue. In this patient no contrast medium was given, but SAx cine images were acquired in full, so exclusion from the study was not necessary.

### MRI characteristics

LV functional analysis revealed that 32% of patients (10/31) had a decreased LVEF below the normal lower limit of 54%. Among all the patients (*n* = 30) who received a Gadolinium contrast agent, LGE was found in 53% (16/30), with myocardial scarring due to ischemia being more common than non-ischemic etiologies (see Table 2).

**Table 2:**
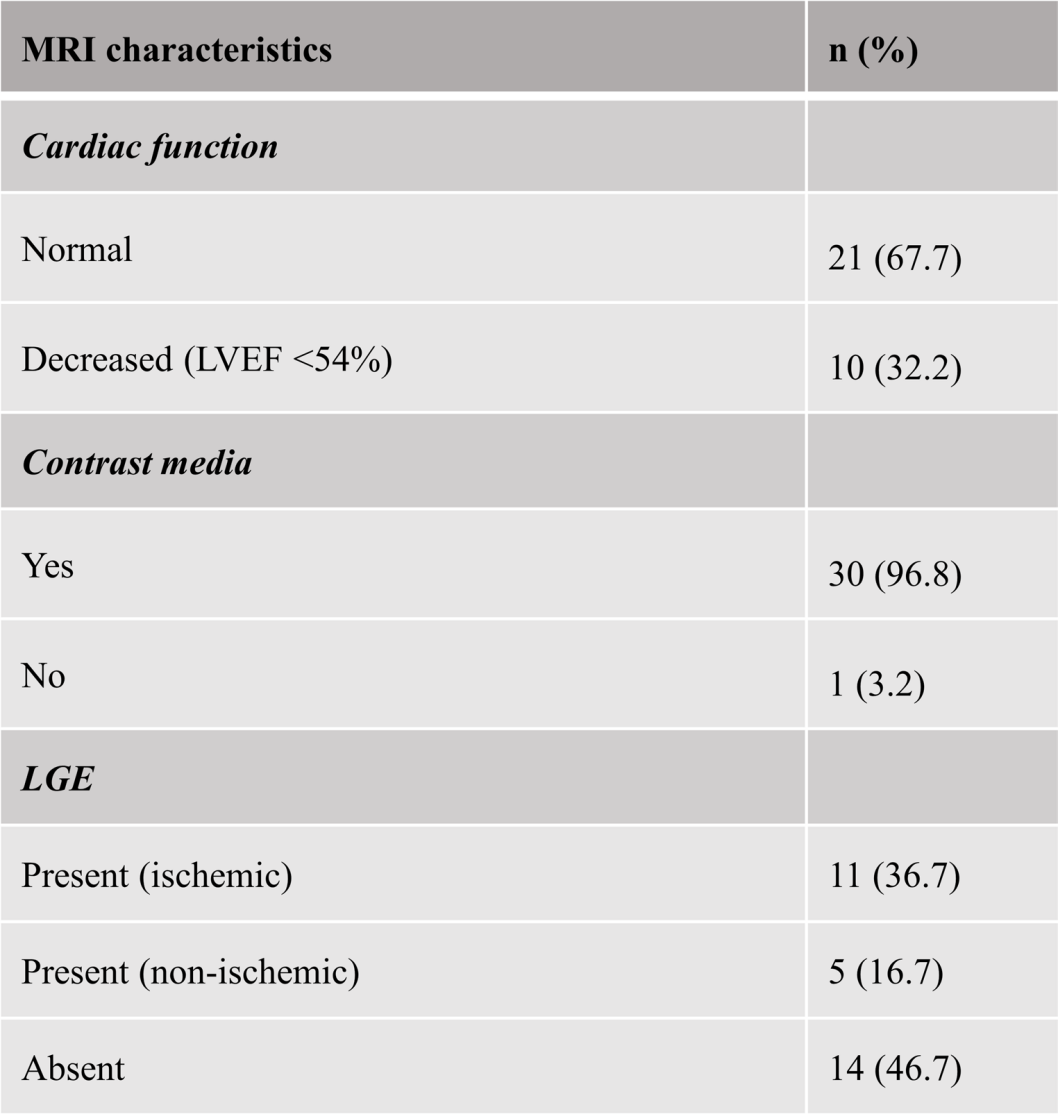
MRI characteristics

### Functional parameters

**Fig. 3:**
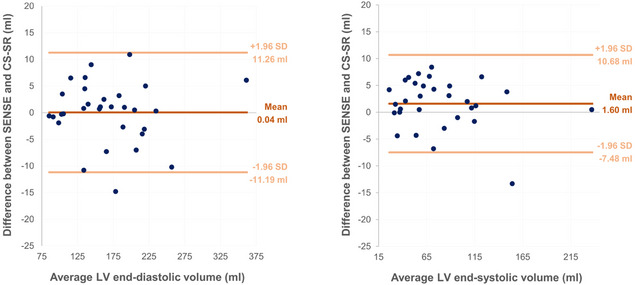
Bland-Altman plot showing the variance in the mean difference in the EDV (left) and ESV (right) between SENSE and CS-SR for each patient for the entire cohort. The mean bias is depicted by the central marking, with the upper and lower markings representing the limits of agreement, and each dot indicating intraindividual measurements

Volumetric analysis revealed no significant differences between the overall mean volumes at SENSE and CS-SR for EDV (SENSE 167.5 ± 58.0 ml, CS-SR 167.5 ± 58.3 ml; t(30) = 0.04, *p =* 0.970) and ESV (SENSE 79.0 ± 45.0 ml, CS-SR 77.4 ± 46.0 ml; t(30) = 1.92, *p =* 0.064). Bland-Altman plots showed a mean difference of 0.04 ml at EDV (LoA − 11.19 ml to 11.26 ml) and 1.60 ml at ESV (LoA − 7.48 ml to 10.68 ml) (see Fig. 3).

**Fig. 4:**
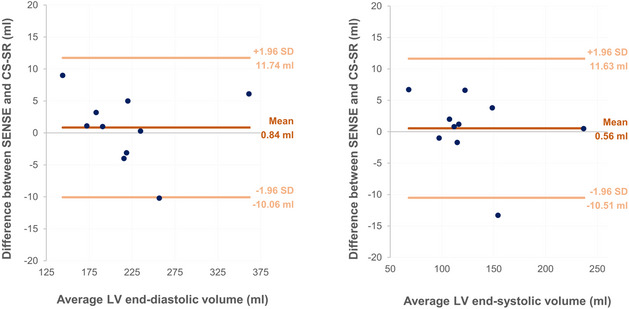
Bland-Altman plot showing the variance in the mean difference in the EDV (left) and ESV (right) between SENSE and CS-SR for a subset of patients with reduced LVEF (< 54%). The mean bias is depicted by the central marking, with the upper and lower markings representing the limits of agreement, and each dot indicating intraindividual measurements

A subset analysis of patients with reduced cardiac output (LVEF < 54%; *n* = 10) revealed no significant differences between SENSE and CS-SR datasets for EDV (SENSE 220.1 ± 59.2 ml, CS-SR 219.2 ± 59.9 ml; *p =* 0.644), ESV (SENSE 127.8 ± 44.7 ml, CS-SR 127.4 ± 46.3 ml; *p =* 0.761), or LVEF (SENSE 42.7 ± 6.2%, CS-SR 42.9 ± 6.2%; *p =* 0.664). Bland-Altman plots showed a mean difference of 0.84 ml at EDV (LoA − 10.06 ml to 11.74 ml) and 0.56 ml at ESV (LoA − 10.51 ml to 11.63 ml) (see Fig. 4).

Interrater agreement (Pearson correlation) was excellent across all the volumetric parameters; EDV: SENSE 0.99, CS-SR 1.00, ESV: SENSE 0.98, CS-SR 0.99.

### Duration of image acquisition and breath-holds

The number of slices acquired per breath-hold was one for SENSE and three for C-SENSE imaging. Total image acquisition time for SAx stacks with whole-heart coverage was significantly longer for SENSE imaging than for C-SENSE imaging (SENSE: 411.1 ± 47.7 s, C-SENSE: 165.6 ± 21.5 s; *p* < 0.001). The duration of breath-holds during image acquisition was significantly longer in C-SENSE acquisition (SENSE: 7.6 ± 1.2 s, C-SENSE: 14.2 ± 1.4 s; *p* < 0.001), with median values of 7 s at SENSE acquisition and 14 s at C-SENSE acquisition.

### Image quality

**Fig. 5:**
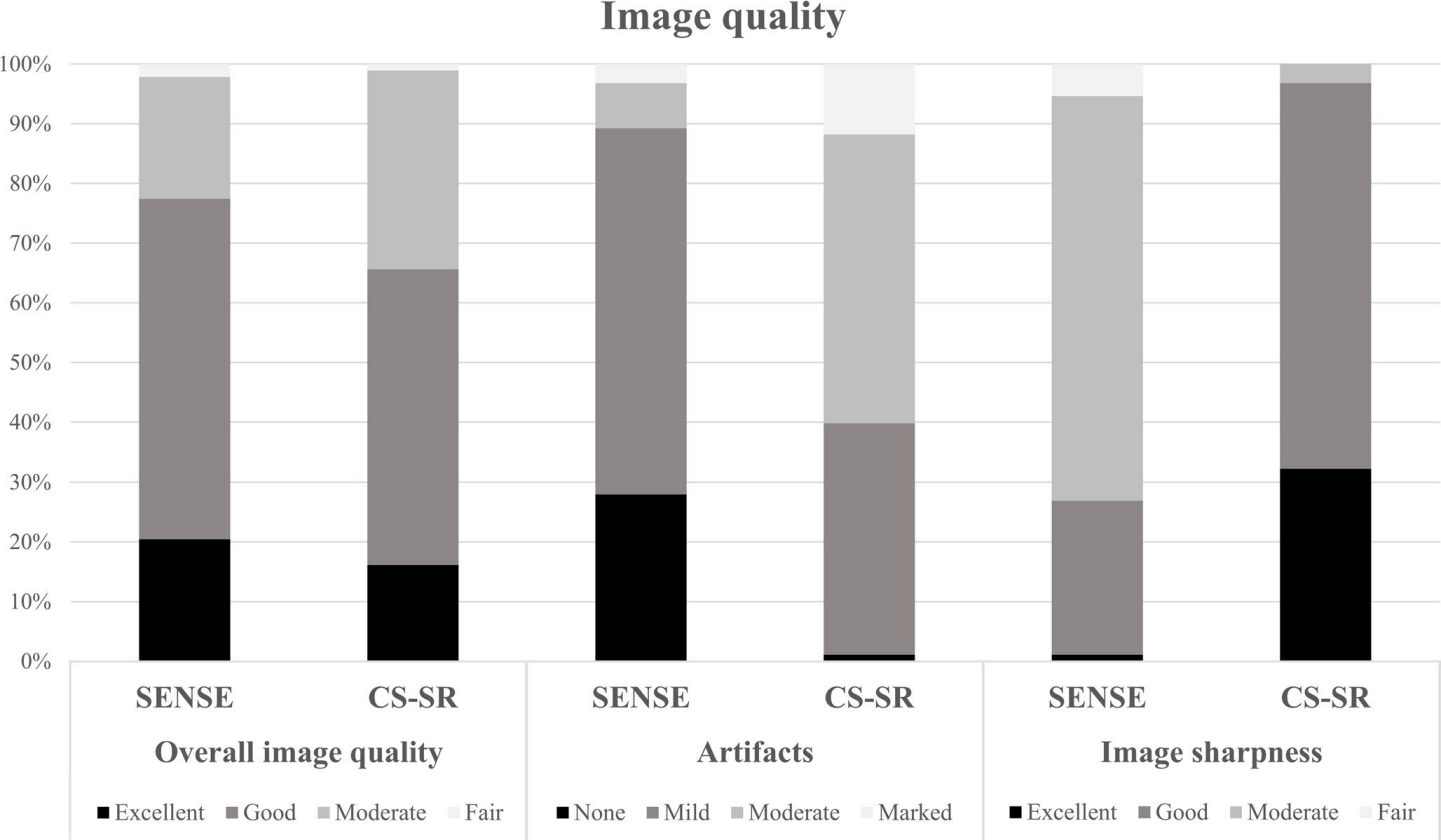
Image quality was assessed using the following subcategories: overall image quality (left), presence and severity of artifacts (middle), and image sharpness (right) for SENSE and CS-SR, respectively

Overall image quality revealed that there were no significant differences between CS-SR and SENSE imaging, where an average reader score of 4 or higher was achieved in 35% (11/31) and 42% (13/31) of patients, respectively (*p* = 0.061) (see Fig. 5).

**Fig. 6:**
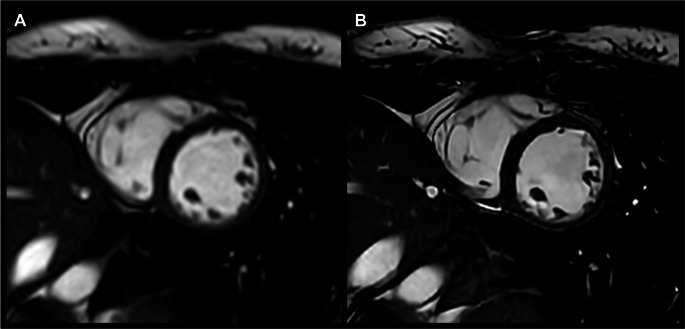
Qualitative assessment of overall image quality at (A) SENSE, *R* = 2 imaging was found to be comparable to (B) CS-SR, *R* = 4 imaging. Increased image sharpness can be noted at CS-SR, notably of the left and right ventricular trabeculations, as observed in this case. No motion artifacts were noted. Supplemental videos (2.1 and 2.2) are available for this case

With respect to image sharpness, CS-SR achieved an average reader score of 4 or higher in most cases, whereas only a small portion of the SENSE images achieved the same score (CS-SR: 52% (16/31), SENSE: 13% (4/31); *p* < 0.001) (see Figs. 5 and 6). The lowest score awarded for both the overall image quality, as well as image sharpness was 2 (“fair”) and the highest was 5 (“excellent”).

**Fig. 7:**
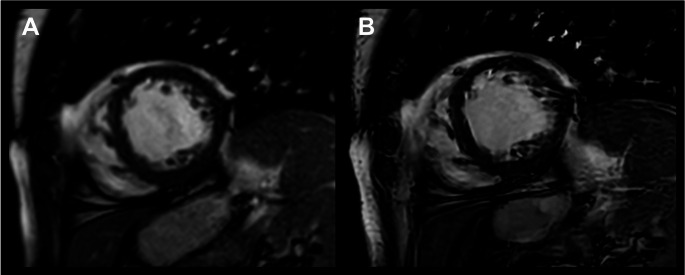
Direct comparison of (A) SENSE, *R* = 2 and (B) CS-SR, *R* = 4 images acquired using an anterior-to-posterior phase encoding direction, at comparable locations and timepoints in the cardiac cycle in the same patient. These images demonstrate intermittently increased respiratory artifacts at CS-SR, which is demonstrated by the double contour of the diaphragm and the blurred contour of the pulmonary vasculature. Supplemental videos (3.1 and 3.2) are available for this case

With respect to the presence and severity of artifacts, SENSE images more frequently achieved an average reader score of 4 or higher, whereas CS-SR images achieved this score less frequently (SENSE: 39% (12/31), CS-SR: 19% (6/31); *p* < 0.001) (see Figs. 5 and 7). The lowest score awarded was 2 (“marked”), the highest was 5 (“none”).

The interrater agreement for the assessment of overall image quality and the presence of artifacts was good (ICC image quality SENSE 0.62 and CS-SR 0.71; ICC artifacts SENSE 0.60 and CS-SR 0.66), and fair with respect to the evaluation of image sharpness (ICC image sharpness ICC SENSE 0.42 and CS-SR 0.41).

### Comparison of quantitative image evaluations

The mean apparent signal-to-noise ratio was significantly lower in CS-SR images than in SENSE images (SENSE: 6.1 ± 4.8, CS-SR: 5.7 ± 4.4; *p* = 0.020). Meanwhile, the mean apparent contrast-to-noise ratio of CS-SR (−13.5 ± 7.2) was comparable to that of standard SENSE imaging (−15.9 ± 9.5; *p* = 0.052). The image contrast showed values of 0.57 ± 0.2 for both SENSE and CS-SR datasets (*p* = 0.96).

## Discussion

Advanced DL-based SR reconstruction algorithms applied to high-resolution, accelerated C-SENSE imaging revealed no significant differences for the quantification of LV function, particularly the volumetric measurements at SAx images, compared with conventional SENSE imaging. CS-SR allowed for significant reductions in scan times, with mean acquisition times shortened from 411.1 ± 47.7 s with SENSE to 165.6 ± 21.5 s with C-SENSE (*p* < 0.001). Although CS-SR improved image sharpness (*p* < 0.001), this benefit was offset by a higher incidence of artifacts (*p* < 0.001).

Cine imaging is an integral part of cardiac MRI and typically accounts for a significant duration of the cardiac MR protocol. In order to shorten overall acquisition times, various image acquisition and reconstruction techniques have been developed. Varied approaches include the integration of parallel imaging and CS with single-shot, single-breath-hold, and real-time image acquisitions, as well as the use of shortened breath-hold durations in combination with low-resolution images, and the upscaling of low-resolution images with CS-SR [11, 15, 16, 34, 35]. The main drawbacks of these approaches include the decreased image resolution commonly found at higher-fold scan time acceleration, blurry contours, and unrealistic breath-hold requirements. We sought to implement a practical solution in routine clinical imaging, by combining C-SENSE with a DL-based SR reconstruction algorithm (Precise-Image-Net) and a denoising filter (Adaptive-CS-Net), in order to maintain comparable image resolution and quality, while reaping the benefits of shortened acquisition times. This technique allowed us to acquire three SAx slices per breath-hold, by use of slightly increased, but well tolerated breath-hold durations.

Several previous studies have shown comparable LV volumetric parameters for SENSE and C-SENSE cine imaging, even in lower-resolution images [11, 15, 36]. Similarly in our study, manual segmentation revealed no statistically significant differences for EDV and ESV across the cohort, with excellent interreader agreement (*r* = 0.97–1.0.97.0). The small intraindividual differences between SENSE and CS-SR, reflected by Bland-Altman limits of agreement of up to 11.26 ml for EDV and 10.68 ml for ESV, are unlikely to be of clinical relevance given that these measurements are themselves subject to inherent intra- and interreader variability. Additionally, no significant volumetric differences were found in a subset analysis of patients with reduced LV function.

Our study focused on maximizing image acquisition speed by increasing the number of slices of high-resolution images acquired per breath-hold, leading to a median breath-hold duration of 14 s. This falls within the lower end of the suggested range for breath-hold durations [37], and was well tolerated by our patients. We observed a notable reduction in acquisition time with C-SENSE compared with standard SENSE imaging, with a mean absolute reduction of approximately four minutes or 60% for a SAx image stack with whole-heart coverage. The conventional SENSE images could have been acquired using two slices per breath-hold instead of one, which would have led to a more comparable duration of breath-holds and a lesser scan time reduction. We decided against this approach, because we wanted to maintain a high-quality benchmark, in order to show the consistency of LV volumetric analysis and more stringently compare the effects on image quality. A drawback is that the confounding effects of scan acceleration and motion cannot be separated, as CS-SR reflects the combined impact of accelerated acquisition (C-SENSE) and DL-based denoising and super-resolution reconstruction algorithms.

The DL-based denoising and super-resolution algorithm was designed to minimize noise and maximize contrast and image sharpness, in order to compensate for the inherently lower image quality found in C-SENSE acquisition. This allowed us to maintain diagnostic accuracy and comparable overall image quality, with identical high-resolution scan parameters. Additionally, CS-SR demonstrated a significant increase in image sharpness, as expected with super-resolution reconstruction techniques [23]. A study by Kravchenko et al., published at the time of writing, applied the same reconstruction methodology to low-resolution C-SENSE images acquired at 3T, comparing them with their standard cine sequences [35]. The authors note that CS-SR appears to improve image sharpness for cine imaging at 3 T, which is consistent with our findings at 1.5T.

In contrast to other studies, we noted an increased presence of motion artifacts in CS-SR imaging compared with our baseline. This may be attributed to the longer breath-hold durations in that acquisition, as this was not a statistically significant finding in studies using shortened breath-hold durations, or studies with cohorts composited of a majority of healthy volunteers with no known history of cardiac disease [11, 35]. Studies of other bodily regions with lower predisposition to motion artifacts, such as the prostate, report fewer artifacts at CS-SR imaging compared to conventional methods [18, 26, 30], which speaks to the robustness of the reconstruction method in general.

Quantitative evaluations revealed a lower apparent signal-to-noise ratio in CS-SR images, whereas the apparent contrast-to-noise ratio was comparable between the two. However, these metrics are limited given the high degree of denoising in the DL algorithms that apply to the area of interest and the reference area.

The main limitations of this study include the small patient cohort, the single-center nature of the trial, and the use of a vendor-specific DL-based reconstruction algorithm. This is the first study of its kind to test this algorithm on a 1.5T scanner. Confirmation of the results with a larger sample size may be warranted. Furthermore, the potential effect of the reconstruction algorithm on regional wall motion abnormalities and the detectability of small anatomic structures, such as clefts or shunts, was not considered during visual analysis.

The possible use of the CS-SR algorithm for other imaging techniques at cardiac MR is intriguing, as the potential reduction in overall acquisition time is surely of interest to most institutions. While opinion leaders in the cardiac imaging community continue to question whether DL-based algorithms are reliable for the use of cardiac MR, this particular reconstruction methodology shows very promising results for cine imaging and demonstrates consistent performance for volumetric parameters analyzed in our patient cohort. While CS-SR can be readily applied to other cardiac MR imaging sequences, future studies should investigate the ramifications on diagnostic utility, for example in T2-weighted imaging or quantitative strain analysis.

In conclusion, CS-SR imaging allows for significantly shortened image acquisition times, without impairing LV volumetric analysis, while preserving overall image quality and resolution.

## Supplementary Information

Below is the link to the electronic supplementary material.

Supplementary Material 1

Supplementary Material 2

Supplementary Material 3

Supplementary Material 4

Supplementary Material 5

Supplementary Material 6

## Data Availability

No datasets were generated or analysed during the current study.

## Abbreviations

MRI: magnetic resonance imaging
ECG: electrocardiogram
bSSFP: balanced steady-state free precession
SENSE: sensitivity encoding
C-SENSE: compressed sensing sensitivity encoding
DL: deep learning
CS: compressed sensing
SR: super-resolution
CS-SR: compressed sensing with super-resolution reconstruction
LV: left ventricle
EDV: end-diastolic volume
ESV: end-systolic volume
LVEF: left ventricular ejection fraction
SAx: short axis
TR: repetition time
TE: echo time
FOV: field-of-view
MR: magnetic resonance
LoA: limits of agreement
ICC: intraclass correlation coefficient
